# Commentary: Hyperoside alleviates myocardial ischemia-reperfusion injury in heart transplantation by promoting mitochondrial fusion via activating the Stat3-Tom70-Opa1 pathway

**DOI:** 10.3389/fphar.2025.1724858

**Published:** 2025-11-21

**Authors:** Qin Yang, Yingjie Liu, Xinzheng Tang

**Affiliations:** Shenzhen Hospital (Futian) of Guangzhou University of Chinese Medicine, Shenzhen, Guangdong, China

**Keywords:** hyperoside, myocardial ischemia-reperfusion injury (MIRI), mitochondrial fusion, oxidative stress, mitochondrial dynamics

## Introduction

The study by Hou et al. (2025) presents a compelling investigation into the cardioprotective effects of hyperoside (Hyp) against myocardial ischemia-reperfusion injury (IRI) in heart transplantation, highlighting the novel Stat3-Tom70-Opa1 pathway as a key mechanism (Murphy and Steenbergen, 2008). This pathway aligns with the established understanding that targeting mitochondrial dynamics, particularly fusion, is a crucial strategy for mitigating IRI, as comprehensively reviewed in the context of melatonin’s protection (Ma et al., 2017). Myocardial ischemia-reperfusion injury (MIRI) is a complex pathological process involving multiple mechanisms such as oxidative stress, calcium overload, and inflammation (Zhang et al., 2024). Against this backdrop, the study by Hou et al. (2025), published in *Frontiers in Pharmacology*, skillfully combines *in vivo* and *in vitro* approaches to demonstrate that Hyp pretreatment significantly attenuates oxidative stress, apoptosis, and inflammatory responses while promoting mitochondrial fusion. The authors’ use of sophisticated techniques such as surface plasmon resonance and cellular thermal shift assays to validate Hyp-Stat3 binding adds considerable mechanistic depth to their findings. However, several aspects of the experimental design and interpretation merit further consideration to strengthen the translational potential of this promising therapeutic approach. This commentary aims to provide constructive feedback regarding methodological refinements and additional experiments that could enhance the impact of this research, particularly focusing on dose-response relationships, control groups, mechanistic validation, and statistical rigor.

A notable opportunity for enhancement lies in the characterization of Hyp’s dose-response relationship. While the authors appropriately selected 100 μM for *in vitro* studies based on preliminary concentration tests, the absence of higher dose groups in both cellular and animal experiments limits the comprehensive understanding of Hyp’s therapeutic window. In mitochondrial-targeted therapies, it is not uncommon to observe bell-shaped dose-response curves where excessive concentrations may lead to paradoxical effects or toxicity, underscoring the delicate balance required in modulating mitochondrial dynamics (Ong et al., 2013). Including additional dose groups, particularly higher concentrations *in vivo* (e.g., 100–150 mg/kg/day), would help determine whether the observed effects plateau or potentially reverse at supraphysiological doses. Moreover, systematic assessment of potential off-target effects through liver and kidney function tests would provide valuable safety data essential for clinical translation. Additionally, exploring dose-dependent effects on Stat3 phosphorylation and mitochondrial fusion markers (e.g., Opa1 cleavage forms) could reveal the mechanistic threshold for Hyp’s efficacy. This would clarify whether higher doses induce saturation or off-target effects, directly informing clinical dosing strategies. Such comprehensive dose-ranging studies would not only establish optimal dosing parameters but also align with FDA guidelines for preclinical drug development.

The absence of recognized positive control groups represents another area for improvement. Without benchmarking against established cardioprotective agents such as MitoQ or other mitochondrial-targeted antioxidants, it becomes challenging to contextualize the magnitude of Hyp’s protective effects within the existing literature. Including such controls would allow for direct comparison of efficacy and mechanism, particularly valuable when novel pathways are proposed. For example, comparing Hyp with MitoQ could highlight differences in targeting mitochondrial dynamics versus general antioxidant effects. If Hyp uniquely upregulates Tom70 via Stat3, it may offer synergistic benefits when combined with existing therapies, enhancing clinical relevance. For instance, demonstrating that Hyp’s protection exceeds or complements that of MitoQ would significantly strengthen the case for its therapeutic potential. Furthermore, the use of additional mitochondrial fusion promoters as positive controls could help validate the specific mechanism proposed by the authors.

The mechanistic evidence supporting the Stat3-Tom70-Opa1 pathway, while innovative, would benefit from additional validation approaches. The current reliance on siRNA-mediated knockdown, though informative, could be complemented by CRISPR-Cas9 knockout models *in vitro* and conditional knockout mice *in vivo* to provide more definitive genetic evidence. Beyond genetic models, incorporating human iPSC-derived cardiomyocytes or *ex vivo* heart perfusion systems could validate the pathway in human-relevant contexts. This step is crucial for translating Hyp into clinical trials, as it addresses species-specific differences. Additionally, while the dual-luciferase assay suggests transcriptional regulation, chromatin immunoprecipitation (ChIP) experiments would directly demonstrate Stat3 binding to the Tom70 promoter, ruling out indirect effects. The statistical analysis description also requires greater transparency, particularly regarding the handling of multiple comparisons. For experiments comparing more than two groups, clear documentation of ANOVA models with appropriate *post hoc* tests is essential to ensure robust conclusions. Providing detailed information about normality testing, variance homogeneity, and specific statistical models would enhance the reproducibility and reliability of the findings. Moreover, emphasizing effect sizes and confidence intervals in future studies could aid in power calculations for clinical trials. This statistical rigor is essential for regulatory approval and ensures that Hyp’s benefits are not overstated in translational research.

## Discussion and future perspectives

The limitations identified above represent opportunities rather than criticisms, reflecting the natural progression of scientific inquiry from initial discovery to rigorous validation. Addressing these considerations would substantially strengthen the foundation for translating Hyp into clinical applications for organ preservation, building upon the well-established mechanisms of IRI (Murphy and Steenbergen, 2008). Future investigations might explore synergistic combinations of Hyp with established interventions such as ischemic preconditioning or machine perfusion protocols, potentially yielding enhanced protection through multi-modal mechanisms. This approach is highly recommended, as recent reviews on MIRI therapeutics emphasize the promise of multi-targeted strategies and drug combinations to achieve superior efficacy (Zhang et al., 2024). The intriguing possibility that the Stat3-Tom70-Opa1 axis may confer protection beyond cardiac transplantation—potentially extending to renal, hepatic, or pulmonary systems—warrants exploration given the universal importance of mitochondrial homeostasis in IRI pathophysiology (Ong et al., 2013).

From a methodological standpoint, incorporating human-relevant models such as induced pluripotent stem cell-derived cardiomyocytes or human explanted hearts would better bridge the translational gap. Additionally, assessing the efficacy of Hyp administration at different time points (e.g., during reperfusion rather than solely as pretreatment) would increase clinical relevance, as practical constraints often limit pretreatment opportunities in transplantation settings. In clinical practice, pre-treating the donor with Hyp is often logistically challenging or impossible. Therefore, exploring alternative administration strategies is crucial for translational potential. For instance, adding Hyp to the preservation solution (e.g., University of Wisconsin solution) during cold storage could be a more feasible approach, allowing the drug to act during the critical ischemia phase. Alternatively, administering Hyp to the recipient upon reperfusion (either systemically or via targeted delivery) could mitigate reperfusion injury. Future studies should directly compare the efficacy of these clinically relevant administration timings with the pretreatment paradigm used in the original study to establish the most effective and practical protocol for heart transplantation. For instance, testing Hyp in large animal models of heart transplantation with prolonged cold ischemia could mimic real-world scenarios. Combining Hyp with machine perfusion protocols may amplify its benefits by maintaining mitochondrial health during organ storage, potentially reducing primary graft dysfunction rates. The authors’ findings open exciting avenues for targeting mitochondrial dynamics as a therapeutic strategy, which is a concept gaining significant traction in IRI research (Ma et al., 2017). With appropriate methodological refinements, this line of investigation holds considerable promise for improving graft outcomes worldwide.

In conclusion, while Hou et al. have made a valuable contribution to the field of cardiac transplantation and mitochondrial biology, fully realizing the potential of their discoveries will require addressing the methodological considerations outlined above. The pathway from promising compound to clinical therapeutic is necessarily rigorous, and each refinement brings us closer to meaningful improvements in patient care. The scientific community would benefit greatly from continued investigation of this pathway with enhanced methodological approaches, potentially revolutionizing how we approach organ preservation in transplantation medicine.

Beyond the mechanistic and methodological considerations, the druggability of Hyperoside warrants discussion. As a naturally occurring flavonoid glycoside, Hyp offers advantages such as a well-defined structure and a history of use in traditional medicine, which may facilitate regulatory approval. However, key challenges remain for its clinical translation. These include its solubility, bioavailability, and metabolic stability, which can influence the effective dose and dosing frequency. Preliminary safety data from this and other studies are promising, but comprehensive toxicological profiling and pharmacokinetic studies in large animal models are essential next steps. Furthermore, the cost-effectiveness of large-scale production and purification of Hyp will be a critical factor determining its accessibility as a potential therapeutic agent for organ preservation. Addressing these druggability aspects will bridge the gap between the compelling preclinical evidence presented by Hou et al. and its future application in clinical trials.
